# Active Collisions in Altered Gravity Reveal Eye-Hand Coordination Strategies

**DOI:** 10.1371/journal.pone.0044291

**Published:** 2012-09-12

**Authors:** Olivier White, Philippe Lefèvre, Alan M. Wing, R. Martyn Bracewell, Jean-Louis Thonnard

**Affiliations:** 1 Unité de Formation et de Recherche en Sciences et Techniques des Activités Physiques et Sportives, Université de Bourgogne, Dijon, France; 2 Institut National de la Santé et de la Recherche Médicale, Unité 1093, Cognition, Action, and Sensorimotor Plasticity, Dijon, France; 3 Institute of Information and Communication Technologies, Electronics and Applied Mathematics, Université catholique de Louvain, Louvain-la-Neuve, Belgium; 4 Institute of Neuroscience, Université catholique de Louvain, Bruxelles, Belgium; 5 School of Psychology, University of Birmingham, Birmingham, United Kingdom; 6 Wolfson Centre for Clinical and Cognitive Neuroscience, Bangor University, Bangor, United Kingdom; Katholieke Universiteit Leuven, Belgium

## Abstract

Most object manipulation tasks involve a series of actions demarcated by mechanical contact events, and gaze is usually directed to the locations of these events as the task unfolds. Typically, gaze foveates the target 200 ms in advance of the contact. This strategy improves manual accuracy through visual feedback and the use of gaze-related signals to guide the hand/object. Many studies have investigated eye-hand coordination in experimental and natural tasks; most of them highlighted a strong link between eye movements and hand or object kinematics. In this experiment, we analyzed gaze strategies in a collision task but in a very challenging dynamical context. Participants performed collisions while they were exposed to alternating episodes of microgravity, hypergravity and normal gravity. First, by isolating the effects of inertia in microgravity, we found that peak hand acceleration marked the transition between two modes of grip force control. Participants exerted grip forces that paralleled load force profiles, and then increased grip up to a maximum shifted after the collision. Second, we found that the oculomotor strategy adapted visual feedback of the controlled object around the collision, as demonstrated by longer durations of fixation after collision in new gravitational environments. Finally, despite large variability of arm dynamics in altered gravity, we found that saccades were remarkably time-locked to the peak hand acceleration in all conditions. In conclusion, altered gravity allowed light to be shed on predictive mechanisms used by the central nervous system to coordinate gaze, hand and grip motor actions during a mixed task that involved transport of an object and high impact loads.

## Introduction

It is well established that eyes and hand are not independently controlled by the central nervous system in experimental contexts like pointing to targets [1], [2], tracking [3], catching a real object [4] or intercepting a virtual ball [5], [6]. This collaborative control also holds in more natural situations like driving [7], tea making [8], or playing table tennis [9], [10]. In most cases, the eyes start moving to the target in advance of limbs (e.g. [11], [12]) suggesting that foveation is crucial for identifying target at high resolution and controlling action [2].

There is an invariant relationship between the spatiotemporal characteristics of eye movements and limb kinematics in goal-directed movements: the end of a saccade toward a target corresponds temporally to the peak acceleration of the hand. Thus, during the decelerating portion of the hand movement, the eye is already on the target and well placed to provide visual information regarding their relative positions for closed-loop control [13], [14].

Despite the underlying importance of vision, only a few studies have examined eye strategies in dexterous manipulations. Pelz and collaborators explored the temporal coordination of eye, hand and head movements while subjects made a sequence of reaching movements to pick up and place blocks in the same configuration as a nearby model [15]. They found that fixations gathered information about the patterns to be reproduced for subsequently guiding hand movements to pick up and place blocks. In another study, participants transported a bar to a target avoiding an obstacle in the direct movement path [16]. Participants almost exclusively fixated certain landmarks critical for the control of the task, like position of potential contact. We can draw two conclusions from the above studies. First, the eye-hand pattern of coordination is stereotyped and the eyes proactively fixate key positions to which the fingertips or grasped object are subsequently directed or avoided. Second, the hand and the moving object are never pursued smoothly by the gaze. Although there is a growing body of work on eye-hand coordination, little is known about the role of gaze to control a hand-held object that contacts a stationary target.

In one notable exception, however, Bowman and colleagues investigated the timing of gaze shifts relative to hand movements in a task in which participants used a mass compensated handle to contact sequentially five virtual objects [17]. The targets, located in the horizontal plane, were always rendered visually and haptically with a robot arm that simulated contact reaction forces. The robot handle was never actuated en route to the targets. In a few trials, the robot motors were turned off and tactile events were prevented. By analyzing these catch trials, the authors found that gaze shifts were programmed and not triggered by tactile feedback related to contact. In that way, the action of the hand around the time of contact is captured in central vision which may facilitate an error estimate by comparing predicted and actual visual consequences of action, and therefore supports error-based learning.

In general, any object manipulation task requires the anticipation of tangential constraints at the fingertips (load force) that must be counteracted by fine adjustment of the grip force (normal to the finger/object interface). For instance, grip force must not only match the tangential force profile when a phone is moved in space but also needs to deal with the contact impulse-like forces induced by the interlocking of the phone in its support base. None of the above studies investigated how eyes, hand and dynamics of prehension are coordinated in a task that involves collisions. Recently, we showed that the central nervous system does not try to predict the exact load force profile that occurs after impact; rather, it applies another predictive strategy consisting in gripping harder about 60 ms after the impact independently of the level of load force resulting from the collision [18]. We showed that this strategy optimizes stability in object manipulation by regulating mechanical parameters including stiffness and damping through grip force.

Here, we analyzed the coordination between gaze, hand and grip force in a collision task in a very challenging dynamical context. Inline with other studies, we expected that eye-hand coordination would show a stereotyped pattern that would however need some time to stabilize given the very novel contexts. Furthermore, the coordination between the oculomotor and grasping systems should be adapted according to the environments to optimize stability at collision. We instructed participants to perform up and down collisions while they were exposed to alternating episodes of microgravity, hypergravity and normal gravity during parabolic flights. We first found that, a comparison between up and down trials in microgravity highlighted a switch in grip force control that occurred at peak hand acceleration. Surprisingly, even after extensive exposure to microgravity where kinematics was symmetric, participants still failed to adjust their grip force accordingly, as reflected by a persistent asymmetry in grip force between up and down trials. Second, we found that durations of fixation increased after impact in micro- and hyper-gravity and in upward trials in every gravitational condition. However, this dwell time did not decrease with practice. Finally, despite dramatic changes in arm movement dynamics and kinematics and gaze asymmetries in the different conditions, we found that the central nervous system invariably triggered a saccade 130 ms before the hand acceleration peaked.

## Methods

### Experimental Procedures

#### Participants

Nine right-handed volunteers (22 to 48 years old, 3 females) participated in the study. Their health was assessed by their various National Centres for Aerospace Medicine as meeting the requirement for parabolic flight. No participant reported sensory or motor deficits. None had previously experienced parabolic flight. All participants gave their written informed consent to participate in this study. The procedures were approved by the European Space Agency Safety Committee, by the Université catholique de Louvain ethics committee and by the French CCPPRB (Comités Consultatifs de Protection des Personnes se prêtant à des Recherches Biomédicales).Two participants experienced nausea during the flight and were not included in the analyses as they did not complete data collection.

#### Manipulation of gravitational context

The experiments took place in the Airbus A300 ZEROg aircraft on three flights from Bordeaux (France) during which a total of ninety parabolas were performed. Thirty parabolas were performed in each flight blocked in three groups of ten parabolas with 2-minute pauses of 1 g-flight between each parabola plus an extra 5 to 8 minutes between each block. A single parabolic flight profile generated a sequence of episodes of normal (1 g), hyper (1.8 g), micro (0 g), hyper (1.8 g) and normal (1 g) gravity of about 20 seconds duration each [19]. Each participant was tested, during the first, second or third block of ten parabolas in one of the flights. Block sequence (1, 2, 3) had no effect on learning on any of the considered variables (all F(2,6)>.56, p>.59); therefore, they were collapsed for the analyses. A three dimensional accelerometer fixed on the floor of the aircraft recorded its acceleration.

#### Task and equipment

The participant was secured by a seat belt on a chair. The task was similar as in our previous investigation (see [18] for details). Briefly, participants produced collisions toward an upper or lower target 45 cm in front of them (30 cm apart from the neutral position) with an instrumented object. The two circular targets (75 mm diameter) were cushioned with 17-mm thick high density foam to limit the impact (stiffness 800 N/m). After contact, they moved the apparatus back to the starting position. An auditory tone prompted movement toward the upper (high tone) or lower (low tone) target and occurred at random between 300 and 500 ms after completion of the previous movement. Five familiarization blocks of 40 randomised up and down collisions were performed prior to the flight. Then each participant conducted the task over 10 complete parabolas. The data analysed correspond to those collected during the first 1 g and 1.8 g phases, plus the 0 g phase, of each parabola. During one parabola, participants typically performed (mean±SD) 9±4, 11±2.9 and 6.3±1.5 trials in normal, microgravity and hypergravity, respectively. Participants were not provided with specific instructions regarding their gaze strategy.

The cylindrical test object (82 mm diameter, 30 mm wide, 212 g mass) was equipped with two parallel force-torque sensors (40 mm diameter, Mini 40 F/T transducers, ATI Industrial Automation, NC, USA) which measured the three force components (F_x_, F_y_, F_z_) along the axes passing through the centre of the corresponding grasp surface. A PXI controller (PXI-8156B) equipped with a 12-bit PXI-6071E analog-to-digital converter (National Instruments, Austin, TX, USA) recorded the synchronized signals from the force sensors at a sampling rate of 1 kHz. In addition, simultaneous horizontal and vertical positions of both eyes were recorded with the Chronos head mounted video-based eye tracker at 200 Hz [20]; CHRONOS VISION GmbH, Berlin, Germany). A second video-based device (OptoTrak 3020 system, Northern Digital, Ontario, Canada) tracked three infrared-light-emitting diodes (IREDs) on the Chronos helmet to reconstruct gaze orientation [21] and three others on the hand-held device. Gaze direction toward the neutral position was oriented slightly downward. The positions of the six IREDs were sampled at a frequency of 200 Hz with a resolution of 0.1 mm within the working environment. Measurements from the eye-tracker, the OptoTrak and the 3 d accelerometer were synchronized.

### Data processing and statistical analysis

Chronos (200 Hz) and OptoTrak data (200 Hz) were linearly interpolated to match 1 kHz (sampling rate of force sensors). The grip force (GF) was calculated as the average of the normal forces applied by the thumb and the fingers on each transducer. The magnitude of the tangential load force (LF) was computed as 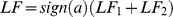 with 
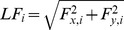
, where F_x,i_ and F_y,i_ are the horizontal and vertical components of the load force of transducer *i* (*i* = 1,2) respectively, and sign(a) is the sign of acceleration. The collision force was obtained by averaging values of 3 samples centred on the maximum of load force.

Saccades were detected using an acceleration threshold of 500deg.s^−2^subject to visual checking. For each trial, we determined the following timings: saccade onset to and arrival on the target (S_onset_ and S_land_, respectively) and saccade leaving the target (S_back_). Duration of target fixation was computed by subtracting the time of saccade landing on the target from the time of saccade onset back to the home position (S_back_-S_land_).

The geometric centre of the manipulandum was calculated from the three IRED positions. Instantaneous velocity and acceleration were obtained using a 5-point central-difference algorithm. Hand movement onset (H_onset_) was defined as the first sample at which the absolute acceleration exceeded 2.5 m.s^−2^ and remained above this value for at least 100 ms. The contact time (T_contact_) was detected backward in time from the highest peak in load force. Kinematic measures of the object included the time of movement onset (H_onset_), peak acceleration (PA) and peak velocity (PV). All trials were aligned at time of impact. We determined the values of grip force and load force at baseline and peak acceleration on individual traces. In addition, grip force at contact (1 ms before the collision) and grip force maximum (GF_M_) were also recorded. This last variable corresponded to the maximum in grip force occurring at least 20 ms after the impact to avoid load force artefacts recorded on the vertical axis induced by small tilts off the vertical plane, as also observed in other studies [22].

Quantile-quantile plots were used to assess normality of the data. A three-way repeated-measure ANOVA was performed on variables of interest to test the effects of direction (Up vs. Down), gravity condition (0 g, 1 g, 1.8 g) and repetition across the ten parabolas. Unless specified otherwise, paired t-test of individual subject means were used to investigate differences between conditions. Alpha level was set to 0.05. The statistical analysis was done using Matlab (The Mathworks, Chicago, IL).

## Results

**Figure 1:**
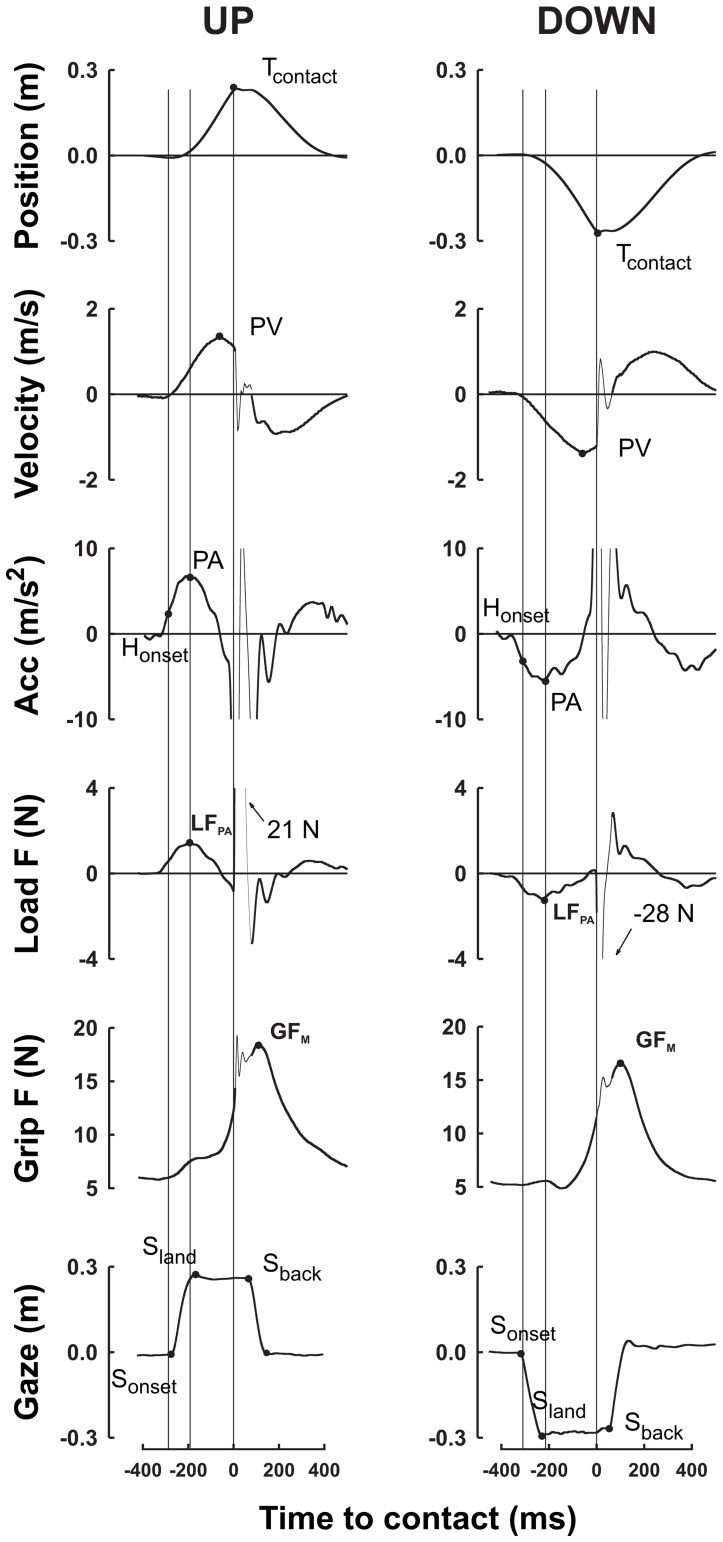
Typical collisions in microgravity. Records of a single collision upwards (left column) and downwards (right column). The following traces are shown as a function of time: vertical position, velocity and acceleration of the object, load and grip forces, and the vertical gaze position. The left, middle and right cursors are aligned with hand onset (H_onset_), peak acceleration (PA) and contact with the target (T_contact_) respectively. PV: peak velocity; LF_PA_: load force at peak acceleration; GF_M_: peak grip force; S_onset_, S_land_ and S_back_: saccade to and arrival on the target, saccade onset back to home position.

Participants performed collisions with an object against two identical targets, located above or below the object's neutral position. The task was conducted in normal (1 g), micro (0 g) and hypergravity (1.8 g). Figure 1 illustrates kinematics, dynamics and gaze movements when the same participant performed collisions to the upper target (UP) and lower target (DOWN) in microgravity. Kinematics is similar as in our previous investigation in 1 g (see [18] for details). A fundamental difference, however, resides in the load force profile. According to Newton's second law, load force (LF) is related to movement acceleration (*a*), mass (*m*) and gravity (*g*) through 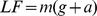. In microgravity (*g = 0*), load force becomes directly proportional to acceleration through mass and simplifies to zero at rest. After movement onset, the load force smoothly follows acceleration during transport of the object (*transport phase*) before increasing sharply to off-scale values at collision (*collision phase*). The grip force (GF) increases as the object approaches the target and reaches a maximum (GF_M_) after the collision. A saccade is triggered to the target at time S_onset_. After a period of fixation (from S_land_ to S_back_), the gaze goes back to foveate the home position.

**Figure 2:**
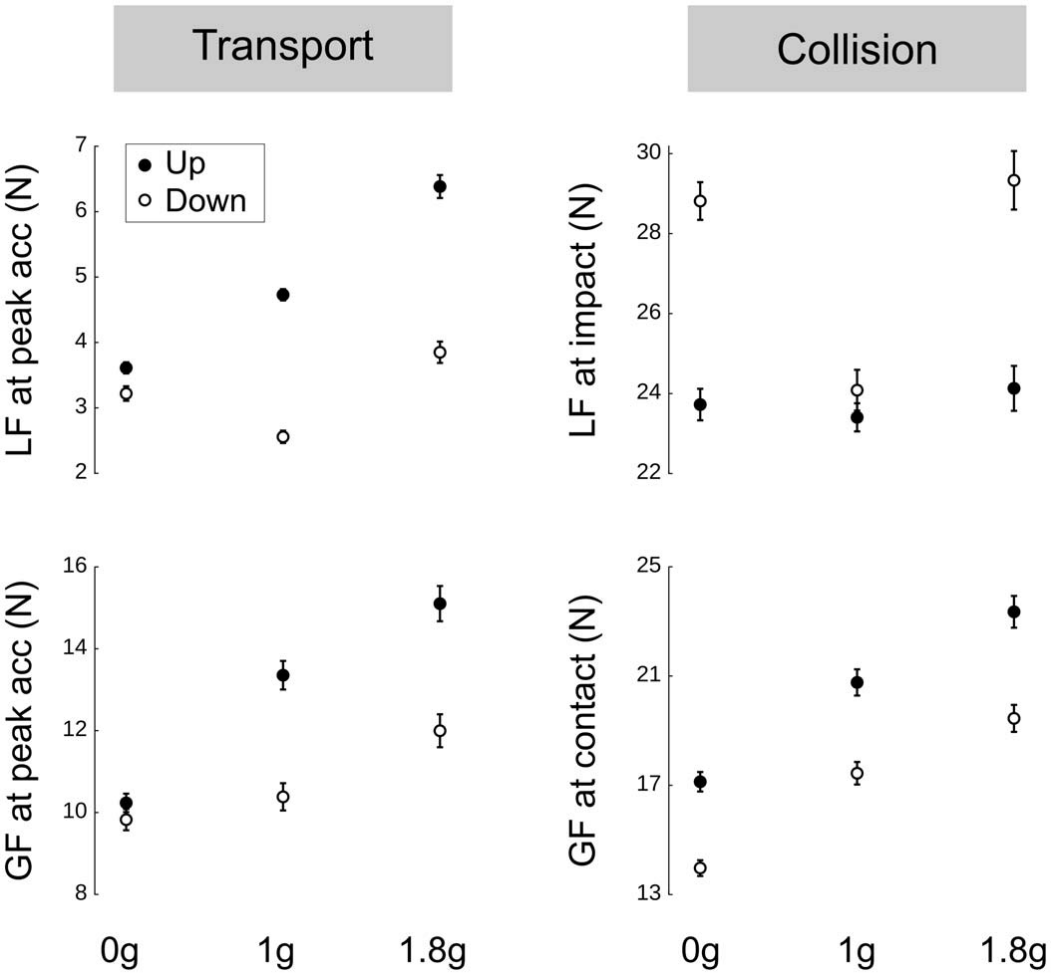
Grip force and load force in the transport and collision phases. Grip force and load force were coupled in the transport phase (left column) but not in the collision phase (right column). Forces are plotted in the two directions (up vs. down) and in three gravity phases (0 g, 1 g and 1.8 g). During transport, both load force and grip force are plotted at peak hand acceleration. At collision, load force is the magnitude of impact and grip force was recorded at contact (8 ms before peak load force), to avoid any artefact. Closed and open disks represent upward and downward movements respectively. Error bars represent between participants SE.

Inertial forces developed during transport to the target are relatively low and distributed over several hundreds of milliseconds around peak of hand acceleration. Figure 2 shows mean load and grip forces at peak acceleration in both directions and in the three gravitational conditions (Fig. 2, left column, Transport). In agreement with previous experiments (see e.g. [19], [23], [24], our data confirm that grip and load forces profiles closely match as quantified by their tight correlation at peak of acceleration in the six direction and gravity conditions (r = .94, p = .006, N = 6). Furthermore, there were reliable within-condition correlations between grip and load forces at peak acceleration in each of the gravity fields (r = .52 to r = .66, all p<.001).

In the collision phase (Fig. 2, right column, Collision), load forces are characterized by large amplitudes and short durations. Participants increased the average level of grip force but did not match the collision forces across conditions (Fig. 2, Collision, upper panel) with grip force (Fig. 2, Collision, lower panel), as quantified by the lack of correlation between impact and grip force at contact (r = −.43, p = .398, N = 6). Participants produced stronger impacts downward (in comparison with upward) in novel environments (0 g: t(6) = 7.82, p<.001; 1.8 g: t(6) = 3.56, p = .012) but similar impacts up and down in normal gravity (t(6) = 1.09, p = .316). In addition, grip force is consistently larger in upper collisions (F(1,36) = 3.4, p = .073) and increases proportionally with gravity level (F(2,36) = 3.38, p = .045). The ANOVA failed to reveal any interaction across direction and gravity conditions (F(2,36) = 0.01, p = .994). Surprisingly, in unfamiliar environments, participants used lower grip force at contact downward compared to upward (0 g: t(6) = −3.25, p<.017; 1.8 g: t(6) = −2.17, p = .073) although they generated stronger collisions to lower targets.

**Figure 3:**
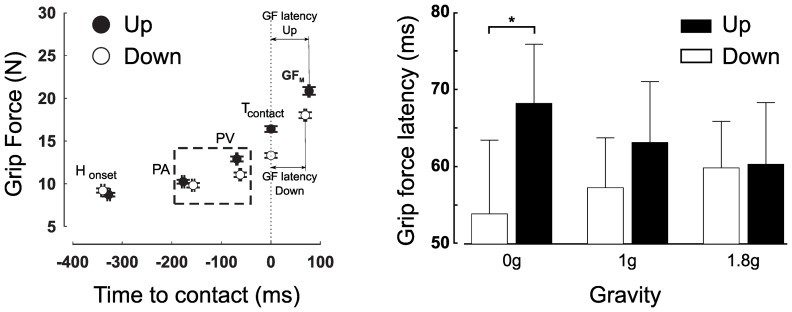
Grip force adjustment over time. (Left) Grip force in function of time to contact in upward (closed disks) and downward (open disks) movements in 0 g. The means and SE of grip forces are reported for the following occurrences: hand onset (H_onset_), peak acceleration (PA), peak velocity (PV), time of contact (T_contact_) and grip force maximum (GF_M_). The horizontal SEs quantify the variability in the occurrences of these events. The vertical dotted line is positioned at contact. (Right) Delay between peak grip forces and contact in upward (black bars) and downward (open bars) trials in 0 g, 1 g and 1.8 g. All values are positive which means that peak grip forces lag the collision. Error bars are between participants SD. Asterisk denotes significant difference (p<0.05).

The control of grip force changes radically from a tight coupling between grip and load forces during the transport phase to shifting peak grip force some 65 ms after impact during the collision phase. Figure 3 (Left) depicts the mean values of grip force and their occurrences at key events during trials performed in microgravity. Participants applied similar grip forces at hand onset (t(6) = −.26, p>.807). Because profiles of acceleration are symmetric in opposite movements (see Fig. 1), load forces at peak hand acceleration reach similar values, and hence, grip forces are also equivalent (Fig. 3, t(6) = −2.01, p = .091). Interestingly, the up and down grip force profiles start to diverge shortly after peak hand acceleration (see frame with grip force remaining larger in upward trials compared to downward at contact (t(6) = −3.25, p<.017) and when it reaches its maximum (t(6) = −2.53, p<.045). Altogether, the comparison between up and down grip force traces in microgravity reveals that the control diverges at the time the object reaches its peak of acceleration (see Figure 3, left panel). Importantly, grip force is not synchronized with contact but peaks 65 ms after (one-sided t-test, t(13) = 9.82, p<.001).

Previously, we demonstrated that peak grip force was invariably delayed after contact and we validated that observation with different target stiffness, directions of movement and catch trials in a virtual environment [18]. We found that this latency was surprisingly robust and insensitive to contextual change. Here, parabolic flights allowed us to challenge the apparent invariability of this latency when participants were confronted with fundamental environmental uncertainties. To quantify this, we measured the latency between peak grip force and contact in the 3 gravity×2 directions. Figure 3 (right panel) shows that this latency is still constant in downward movements (pairwise comparisons across 0, 1 and 1.8 g, all t(6)<1.98, p>.095). In microgravity, participants exerted peak grip force 14 ms later in upward movements compared to downward (27% increase, t(6) = 2.54, p = .044). This significant difference disappeared in the last 3 parabolas (t(6) = −.29, p = .782). No other differences were found in normal and hypergravity. In sum, the latency between peak grip force and contact was significantly longer during upward movements in microgravity, which stresses the particularity of this environment.

**Figure 4:**
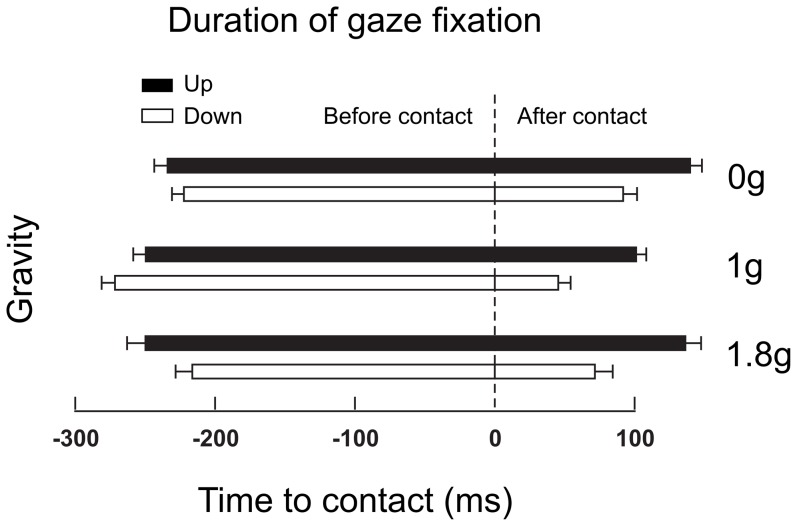
Durations of target fixations before and after target contact. Saccades landed on the target 241.1±84.1 ms before the collision but left later in upward compared to downward trials (black bars vs. open bars) and in unknown gravity fields (0 g and 1.8 g vs. 1 g). The vertical dashed line denotes time of contact. Error bars are between participants SD.

When generating collisions, collection of information is essential to maintain a control on the object; this need is enhanced in novel gravity conditions. We now focused our analyses on durations of target fixation as a function of time of contact. Figure 4 presents the duration of target fixation before and after contact. Participants produced a saccade to the target such that the eyes arrived at the target on average 241.1±84.1 ms before the collision. This timing is invariant across conditions; the ANOVA does not report any significant main effect or interaction (all F<.58 and p>.116). In contrast, the duration of foveation of the target is longer *after* contact in upward movements (main effect of direction, F(1,36) = 11.8, p = .002) and in novel gravity fields (main effect of gravity condition, F(1,36) = 3.26, p = .044). Post hoc t-tests show that durations of fixation are the shortest in normal gravity compared to microgravity (t(6) = −4.52, p = .004) and hypergravity (t(6) = 3.13, p = .020). The interaction is not significant, (F(1,36) = .17, p = .844) the directional increment in target fixation being equivalent in the three gravity levels (56 ms). However, the duration of this post-impact fixation is longer in the more challenging conditions (up, and novel gravity). Interestingly, this duration of fixation does not decrease with repetition of the task (first three vs. last three parabolas: t(6) = 1.1, p = .303)

**Figure 5:**
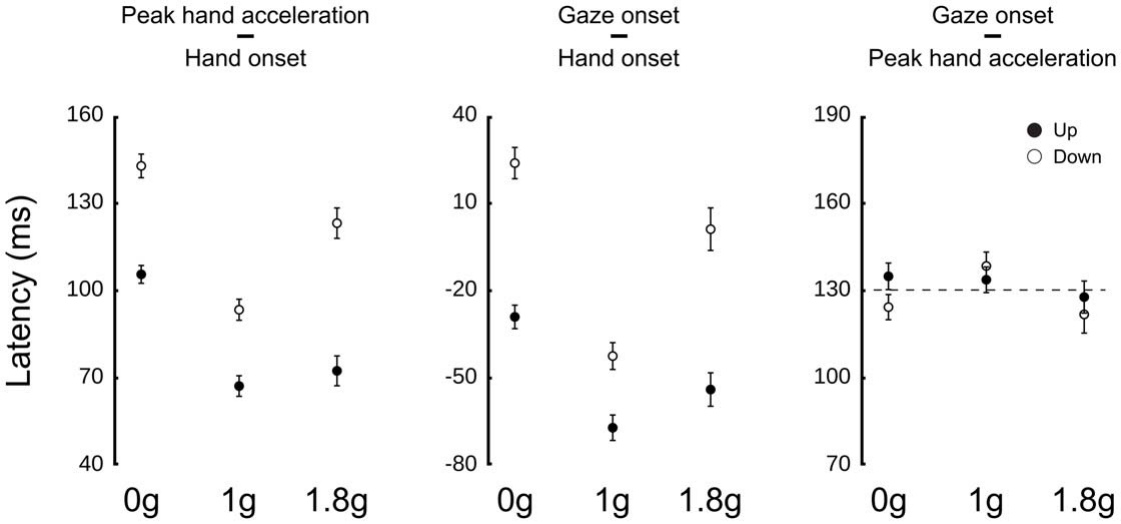
Constant latency across direction and gravitational conditions between times of peak hand acceleration and gaze saccade onset to the target. Relative latencies between peak hand acceleration and hand onset (left panel), gaze onset and hand onset (middle panel) and between gaze onset and peak hand acceleration (right panel) in the three gravity fields. Open and closed disks denote downward and upward trials, respectively. Error bars represent between participants SE. The horizontal dashed line in the right panel is positioned at mean latency (132.6 ms).

Previous studies have reported strong relationships between gaze and hand movements, namely, between the end of a saccade and peak hand acceleration [14]. Altered gravity induced a lot of variability in arm movement timing kinematics and dynamics, allowing us to ask, to what arm movement features eye movements are coupled. For instance, we observe increased hand reaction times upward in all gravity conditions. This holds true in 1 g (t(6) = −2.4, p = .047) and 1.8 g (t(6)<−3, p<.023) but also in 0 g, despite the absence of gravity (t(6) = −4.7, p = .003). Consequently, hand acceleration does not peak at the same time after hand onset. Figure 5 (left panel) illustrates this observation and shows that peaks of acceleration are significantly influenced by direction (F(1,36) = 19.5, p<.001) and gravity (F(2,36) = 11.7, p<.001). Similarly, Figure 5 (middle panel) also shows that, overall, saccades are triggered 35 ms before hand onset but that this timing is highly dependent on direction (F(1,36) = 15, p<.001) and gravity conditions (F(1,36) = 8.8, p = .001). However, when we consider gaze latencies relative to peak hand acceleration (Fig. 5, right panel), we find remarkably constant timings: saccades are initiated on average 132.6±83.5 ms before the hand reaches its peak of acceleration, regardless of direction or gravity (no difference across conditions, all F<0.7, p>.504). Further analyses demonstrate that this time-locking does not hold for any other kinematic characteristics.

**Figure 6:**
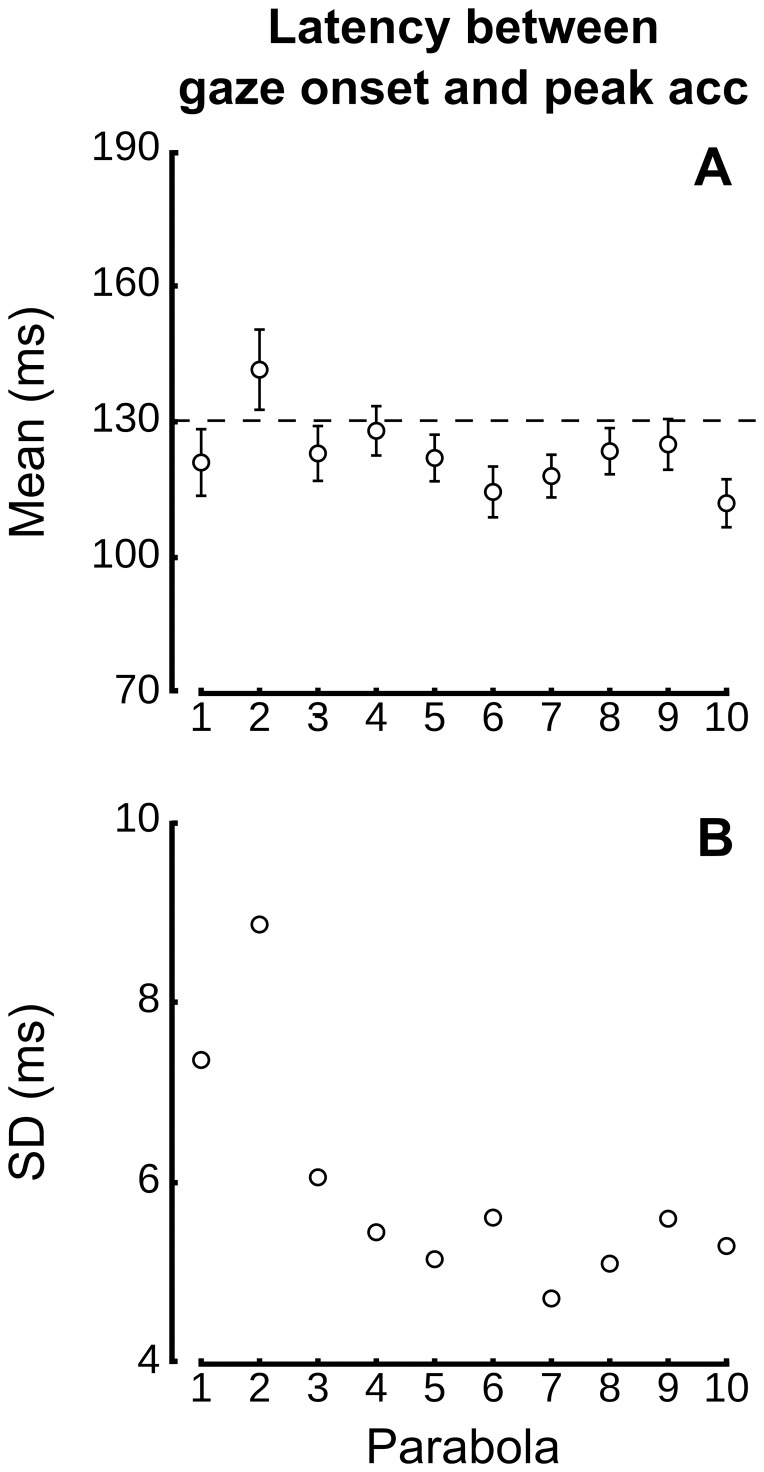
Decrease of variability of latency between times of peak hand acceleration and gaze saccade onset to the target across parabolas. Mean gaze latencies relative to peak hand acceleration (A) and standard deviations of mean latencies (B) across the 10 parabolas. The horizontal dashed line in the top panel is positioned at mean latency (132.6 ms). Panel B reports a strong decrease in variability within three parabolas.

At this point, it is interesting to ask the question whether this invariant timing between peak hand acceleration and saccade onset is established from the very first trials or, rather, is a process that converges to a fixed latency? Figure 6 depicts that the latency does not vary across the 10 parabolas (panel A). In contrast, Panel B shows that an important decrease in variability, as measured by SD of latencies, occurs within 3 parabolas. A repeated-measures ANOVA across parabolas quantifies this observation and does not report any significant effect on mean latency (F(9,54) = 0.4, p = .924), but highlight a strong decrease in variability (F(9,54) = 2.3, p = .027). A post hoc t-test reveals that SDs of latencies decrease significantly between parabolas #1–2 and parabolas #3–10 (t(6) = 2.6, p = .039). Although the set point is established from the very first episodes of altered gravity, trials are needed to decrease variability.

## Discussion

We analyzed gaze-hand-grip coordination when participants controlled collisions against fixed targets in different gravitational environments. This allowed us to test the importance of gaze in learning and how eyes and hand were coordinated. First, grip forces followed load force profiles during the initial transport phase; grip force was then decoupled from load force and increased rapidly in approach to the target, which marked a qualitative transition between the two predictive modes of control. The maximum of grip force occurred only 65 ms after collision. Microgravity revealed that the central nervous system changed its mode of predictive grip force control within a trial, around the time of peak hand acceleration. Second, participants produced longer fixations of the target after contact in the more challenging conditions (up, and novel gravity). Third, saccades onsets were time-locked to the peak hand acceleration in all conditions, despite great variability in hand kinematics.

### Microgravity reveals the time course of two predictive grip force mechanisms

In the transport phase, the tight coupling between grip and load forces further confirms anticipatory control of grip force, whatever the direction of movement and gravity level, and in a task involving both transport and collisions. This is in agreement with previous studies obtained in altered gravity where participants adjusted their grip force according to the load force in vertical point-to-point [25], [26] and rhythmic movements in steady gravity phases [23], [19] and gravity transitions [27]

Microgravity allowed us to unravel how the central nervous system controls grip force so differently in the transport and collision phases. Upward and downward peaks of acceleration, and therefore associated load forces, were symmetric around zero (transport phase) but impacts were larger downward (collision phase). Nonetheless, grip forces diverged after the hand reached its peak acceleration, as clearly shown by superimposing up and down grip force traces. This elegantly identifies a transition between the two phases. Indeed, since load force decreased between peak acceleration and contact in both directions (see load force in Fig. 1), the increase in grip force can be explained by a change in the strategy in anticipation of the collisions.

Grip force in the collision can be regulated by high level and low level mechanisms. First, the absolute magnitude of grip force can be adjusted according to the global task context, providing an adequate safety margin. For instance, in an aircraft where perturbations are more likely than on the ground, a passenger will increase her/his level of grip force to hold a cup of coffee. In altered gravity, we consistently observed larger collisions downwards but larger grip force upwards. Interestingly, other investigations suggested that the stimulus that challenges grasp stability (increased load) is of greater practical relevance compared to the stimulus that does not challenge grasp stability (decreased load) [28], [29]. Thus, a larger safety margin in upward collisions may be a good strategy to cope with higher risks of dropping the object. However the question remains as to why this asymmetry persists in microgravity, where potential slips are equally probable in both directions? We speculate that with extensive training in long term weightlessness, astronauts would eventually produce similar impacts and grip forces in both directions, as it is the case in horizontal collisions [17], [30], [31], [32]. Thus, internal models incorporate the action of gravity to adjust the motor plan in simple tasks [25], [33], [19] but we suggest that more time is needed to converge to an optimal behaviour in tasks of higher complexities. Our results also further emphasize the capacity of the Central Nervous System to adapt to the massive changes of the environmental conditions.

At a lower level of control, the delay between impact and peak grip force is an important parameter that optimizes mechanical properties and object stability at the collision. We previously showed that this latency was very robust to changes in experimental contexts [18]. However, profound gravitational changes induce significant modulation of that latency. In the particular condition of upward movements in microgravity, grip force peaked later than in any other condition. However, the increment initially present disappeared in the last parabolas. We hypothesize that this strategy is coherent with the high uncertainties associated with early upward trials in 0 g. By shifting even more the occurrence of the peaks grip force, participants allowed the springiness properties of the finger/object interface to dissipate more energy, and therefore improved stability.

### Gaze maintains an optimal working memory

Gaze acquired the target some 241 ms before the hand did, and maintained fixation for 50 to 150 ms after collision. Although the dwell before contact was insensitive to conditions, it varied substantially after the object collided with the target. Foveation of the contact allows comparison of predicted and actual consequences of the action. We suggest that this strategy permits the sensorimotor system to establish a mapping between retinal and extra retinal signals and other sensory signals including information coming from mechanoreceptors that arise from contact. In that way, the working memory of previous successful trials can be updated. Longer durations of fixation after contact in unknown gravity fields and upward movements strengthen this assumption: More time is spent to refine the correlation between actions and their consequences [34], [35]. Furthermore, retrieving information from working memory where visual information was encoded foveally has been shown to be optimal in speeded tasks [36], compatible with straight collisions. Finally, durations of fixation did not decrease with trials. This supports the idea that the role of visual feedback is not diminished with practice. Instead, participants learn to make better use of visual information and use online processing to optimize actions toward the task goals [37].

### The ocular motor system monitors the efference copy of the arm motor command

Participants invariably triggered a saccade to the target 130 ms before the object reached its peak acceleration, in all conditions. This strategy provided visual feedback of the hand approaching the target in the slowing down part of the movement [2]. In another study examining gaze-hand coordination in visually guided pointing, Neggers and Bekering showed that preparation of saccades was initiated about 170 ms before the fingertip contacted a target [17], [38], [39]. Importantly, in addition to supporting a predictive gaze strategy, these findings also suggest that the oculomotor system can directly use an internal signal related to the arm motor command rather than visual information related to the scene [40], [41].

Here, we examined for the first time the coordination of three apparently distinct modalities in the same task: gaze, hand and grip force controls. Our results strongly suggest that the occurrence of peak hand acceleration provides information that allows the tight coordination of the motor system, key to achieve the task successfully.

First, the occurrence of peak hand acceleration marks the transition between transport and collision phases in grip force control. On the basis of an efference copy of the arm motor command, a forward model predicts the load force that will be generated at the fingertips as a consequence of kinematics and gravitational condition. This forward model has been shown to encode gravity [25], [33], [42]. Indeed, two equivalent motor commands will induce different load forces at the fingertips in, say, 1 and 1.8 g environments. The function of this forward model is to predict the required grip force according to the phase of the movement, *i.e.* transport or collision.

Second, the visual system monitors the efferent copy of the arm motor command and launches a saccade 130 ms before the arm reaches its peak acceleration. Strikingly, this latency was constant from the very first parabolas, despite considerable differences (up to 60 ms) in the timing of arm movements and saccade asymmetries between up and down directions. This particular result suggests that saccade onset – and less saccade landing - may be a variable worth considering when investigating oculo-manual tasks. Furthermore, although the system was ‘calibrated’ from the first trial it needed two episodes to stabilize its variability down to an acceptable level. Two parabolas are roughly equivalent to 45-second and 20-second exposures to microgravity and hypergravity, respectively, which represent about 22 and 12 trials in 0 and 1.8 g. Taking the time needed to prepare and trigger the saccade into account which is about 170 ms [43], this means that the oculomotor system can predict when the hand will reach its peak of acceleration up to 300 ms in advance. The Central Nervous System also chooses to time-lock a saccade such that visual feedback will be prevented during the portion of the movement the vision is the poorest to collect information, i.e. when acceleration is maximal [44]. Such powerful predictive mechanisms may be at the cost of increased preparation times. Indeed, we reported longer hand reaction times in upward movements in all gravitational conditions, including in weightlessness. However, this particular condition clearly rules out a possible neuromuscular delay due to an increased gravitational torque upward, because hands also departed later in 0 g. We therefore suggest that these longer latencies in upward movements reflect extra processing time required for the planning of the upcoming task in feedforward manner.

Although contact events give rise to salient information from different modalities (such as audition, vision and proprioception), our results altogether suggest that peak of hand acceleration provides a robust unbiased central signal for intermodal alignment.
